# Optimizing clinical guidelines to address antimicrobial-resistant infections: A conceptual framework reflecting stakeholder perspectives

**DOI:** 10.1017/ash.2022.319

**Published:** 2023-02-16

**Authors:** Rhea E. Powell, Sue Felt-Lisk, Arnold Chen, Casey Sullivan

**Affiliations:** 1 Mathematica, Washington DC; 2 Office of the Assistant Secretary for Planning and Evaluation, US Department of Health and Human Services, Washington, DC

## Abstract

**Objectives:**

Clinical guidelines or guidance is an important tool for preventing and treating antimicrobial-resistant (AMR) infections. We sought to understand and support the effective use of guidelines and guidance for AMR infections.

**Methods:**

Key informant interviews and a stakeholder meeting on the development and use of guidelines and guidance for management of AMR infections; the interview findings and meeting discussion informed a conceptual framework for AMR infection clinical guidelines.

**Participants:**

Interview participants included experts with experience in guidelines development and physician and pharmacist hospital leaders and antibiotic stewardship program leaders. Stakeholder meeting participants included federal and nonfederal participants involved in research, policy, and practice related to prevention and management of AMR infections.

**Results:**

Participants described challenges related to timeliness of guidelines, methodologic limitations of the development process, and issues with usability across a range of clinical settings. These findings, and participants’ suggestions for mitigating the challenges identified, informed a conceptual framework for AMR infection clinical guidelines. The framework components include (1) science and evidence, (2) guideline and guidance development and dissemination, and (3) implementation and real-world practice. These components are supported by engaged stakeholders whose leadership and resources help to improve patient and population AMR infection prevention and management.

**Conclusions:**

Use of guidelines and guidance documents for management of AMR infections can be supported through (1) a robust body of scientific evidence to inform guidelines and guidance; (2) approaches and tools to support timely, transparent guidelines that are relevant and actionable for all clinical audiences; and (3) tools to implement guidelines and guidance effectively.

Antimicrobial-resistant (AMR) infections pose a serious risk to health, with >2.8 million cases resulting in 35,000 deaths each year in the United States.^1^ Although infection control and prevention efforts have contributed to reduced AMR infections and deaths in recent years, AMR infections and deaths remain a pressing challenge in health care and community settings. Strategies to mitigate AMR infection risk include infection prevention efforts in health care and community settings, early detection, and response to contain emerging threats, as well as improving appropriate antibiotic use. Clinical guidelines provide recommendations for evidence-based care, informed by a systematic review of evidence and an assessment of the benefits and harms of care options.^2^ They are important tools to help prevent and manage AMR infections.^3,4^ Standards for guideline development, such as the Institute of Medicine *Clinical Guidelines We Can Trust*, have set norms for establishing transparency, managing conflicts of interest, selecting the development group, reviewing evidence, and articulating recommendations, as well as conducting external review and updating.^2^ Additionally, the Grading of Recommendations Assessment, Development, and Evaluation (GRADE) approach has been developed to ensure that guidelines provide recommendations based on rigorous evidence.^5,6^ Yet developing and updating clinical guidelines and translating them to clinical practice remain challenging. Practicing clinicians do not consistently use clinical guidelines to inform their decision making^7^; thus, approaches to improve the development and usability of guidelines are needed.^8^

In 2020, the Office of Science and Data Policy within the Office of the Assistant Secretary for Planning and Evaluation (ASPE) within the US Department of Health and Human Services (HHS) and the Food and Drug Administration (FDA) funded a project to assess (1) the current process for developing and updating clinical practice guidelines and guidance documents for the treatment of infectious diseases, including AMR infections; (2) how infectious disease guidelines and guidance inform treatment strategies in practice; and (3) key informant suggestions for supporting effective guidance for AMR infections. To address these questions, we performed key informant interviews and facilitated a stakeholder meeting focused on the development, dissemination, and implementation of guidelines and guidance.

## Methods

### Key informant interviews

We conducted key informant interviews with 8 infectious diseases experts with experience in guideline development, 6 hospital leaders, and 7 pharmacists involved in hospital or health-system antimicrobial stewardship programs. We identified interview participants through reviewing information such as authorship of guidelines or guidance on related initiatives and potentially relevant organization websites, and the membership of the Presidential Advisory Council on Combating Antibiotic-Resistant Bacteria. Interviews with key informants covered a range of topics including (1) perspectives on the patterns of antimicrobial resistance in hospital and community settings; (2) awareness of the Infectious Disease Society of America (IDSA) guidance on AMR gram-negative infections^3,4^ and views of different types of guidance; (3) structure and process for making formulary decisions and the role of guidance in those decisions; (4) effects of new guidance on hospital operations; (5) internal versus external guidelines or guidance; (6) prescribing patterns of antimicrobial drugs; (7) antimicrobial stewardship program activities; and (8) how guidelines or guidance for hospitals can be made most helpful (generally or specifically for AMR infections). A transcriptionist transcribed all interviews and the research team qualitatively analyzed transcripts to identify key themes.

### Stakeholder meeting

Building on the findings from the key informant interviews, we convened key stakeholders to discuss ways to improve the development and implementation of AMR infectious disease clinical practice guidelines. The meeting was facilitated virtually in March 2022 during 2 consecutive half-day sessions. More than 50 participants representing both federal and nonfederal organizations participated, including clinicians, scientists, government officials, and other experts involved in guideline or guidance implementation or related research. Federal participants included representatives engaged in related work within the Agency for Healthcare Quality and Research (AHRQ), ASPE, Biomedical Advanced Research and Development Authority (BARDA) within the Office of the Assistant Secretary for Preparedness and Response, Centers for Disease Control and Prevention (CDC), Centers for Medicare & Medicaid Services (CMS), FDA, and National Institutes of Health (NIH), all agencies within HHS. During the meeting, several presenters shared work relevant to developing AMR infection guidelines and implementing best practices to mitigate antimicrobial resistance. For example, presenters shared lessons learned from developing COVID-19 guidelines on a rapid timeline and findings from studies of antibiotic stewardship interventions. Meeting participants also engaged in small group breakout sessions to discuss the topics presented and key questions. A note taker in each main session and breakout session documented themes that emerged.

## Results

Key informants and stakeholder meeting participants identified challenges related to the development and use of guidelines for AMR infection in clinical practice (Table 1).

**Table 1.** Challenges in Development and Use of Guidelines and Guidance

Challenges Related to Guideline Development	Challenges Related to Implementation and Use
Timeframes for development that are so long that the resultant guidelines may be outdated by the time they are published	Guidelines and guidance cannot be applied appropriately in diverse clinical settings
Issues with the GRADE method’s criteria, which do not weight studies appropriately in the context of AMR	Challenges in educating clinicians on the guidance, and reaching different types of clinician audiences
Lengthy final documents that make it difficult for busy clinicians to locate key information	Difficulties or delays in making changes to electronic health records that would help with adherence to guidance

Note. GRADE, Grading of Recommendations Assessment, Development, and Evaluation; AMR, antimicrobial resistance.

### Timeliness of guidelines

The long timeframe for developing guidelines is one challenge. Specifically, the numerous steps required by the IOM and GRADE process are time consuming, including time needed to formulate the questions for the literature searches, to conduct the literature search, to discuss and agree on recommendations, and then to finalize and write guideline recommendations. One interview participant said about the guideline development process, “… A formal guideline has taken years to do … and in a dynamically changing environment, like bacterial pneumonia, they’re almost out of date by the time that they get published … the bacteria are changing and developing resistance patterns while the antibiotic armamentarium may be changing as well. And antibiotic resistance patterns are changing, including to the novel antibiotics.”

The time required to develop guidelines can be exacerbated by the difficulties of assembling mostly volunteer panelists to complete their assignments on time or to reach agreement on issues. One interview participant with experience developing antibiotic stewardship program guidelines estimated that ∼40 hours of calls were held over several weeks throughout the course of guideline development. The lengthy process for developing guidelines can sometimes mean that, by the time guidelines are published, they already need updating.

### Methodological criteria may limit clinical relevance

Some interview and stakeholder meeting participants noted that the GRADE methodological criteria may limit consideration of relevant evidence in guideline development. GRADE’s stringent methodological criteria may place too much emphasis on rigorous but less clinically relevant studies and too little emphasis on studies that have clinical importance but are less methodologically rigorous. For example, several interviewees with experience developing guidelines noted that clinical trials conducted by pharmaceutical companies for FDA approval of new antimicrobials are often of high quality by GRADE criteria, but because their goal is to demonstrate efficacy of the new drug, they may minimize variability in enrolled patients and test situations that may not always be relevant in clinical practice. The trials may exclude patients infected by highly resistant organisms or compare the trial drug against a control drug that most practitioners would not have chosen. In short, the approach may overemphasize methodological study quality at the expense of clinical judgment and expertise.

### Length and usability of guidelines and guidance

Final published guidelines are frequently large, detailed documents. Interview and stakeholder meeting participants noted that guidelines can be unwieldy for busy clinicians to navigate. As one interview participant said, “[Guidelines] are very long documents, they have a lot of heft and weight behind them and all that great authority that comes with having such well-referenced, evidence-based guidelines. But people in the front lines are really looking for something that was a little bit more digestible, usable.”

### Implementing guidelines and guidance in different settings

Key informants cited challenges related to implementing guidelines across a range of settings and with different clinical audiences. Respondents cited variation in the prevalence of AMR infections across different healthcare settings, noting that although most tertiary-care medical centers see significant AMR infections, community hospitals may manage such infections to varying degrees and may need to address them with varying resources. Although antibiotic resistance has improved overall since 2013, it has worsened in nonhospital community settings,^1^ which may experience different challenges to implementing AMR infection guidelines. Interview and stakeholder meeting participants noted that an important source of AMR infections is long-term care facilities, which typically do not have their own laboratories to track this problem. Participants also cited difficulties or delays in making changes to electronic health records (EHRs) to align with clinical guidelines, as well as challenges educating clinicians on guidelines and guidance.

## Addressing challenges to guidelines: A conceptual framework for guidelines and guidance for the treatment of AMR infections

**Fig. 1.**
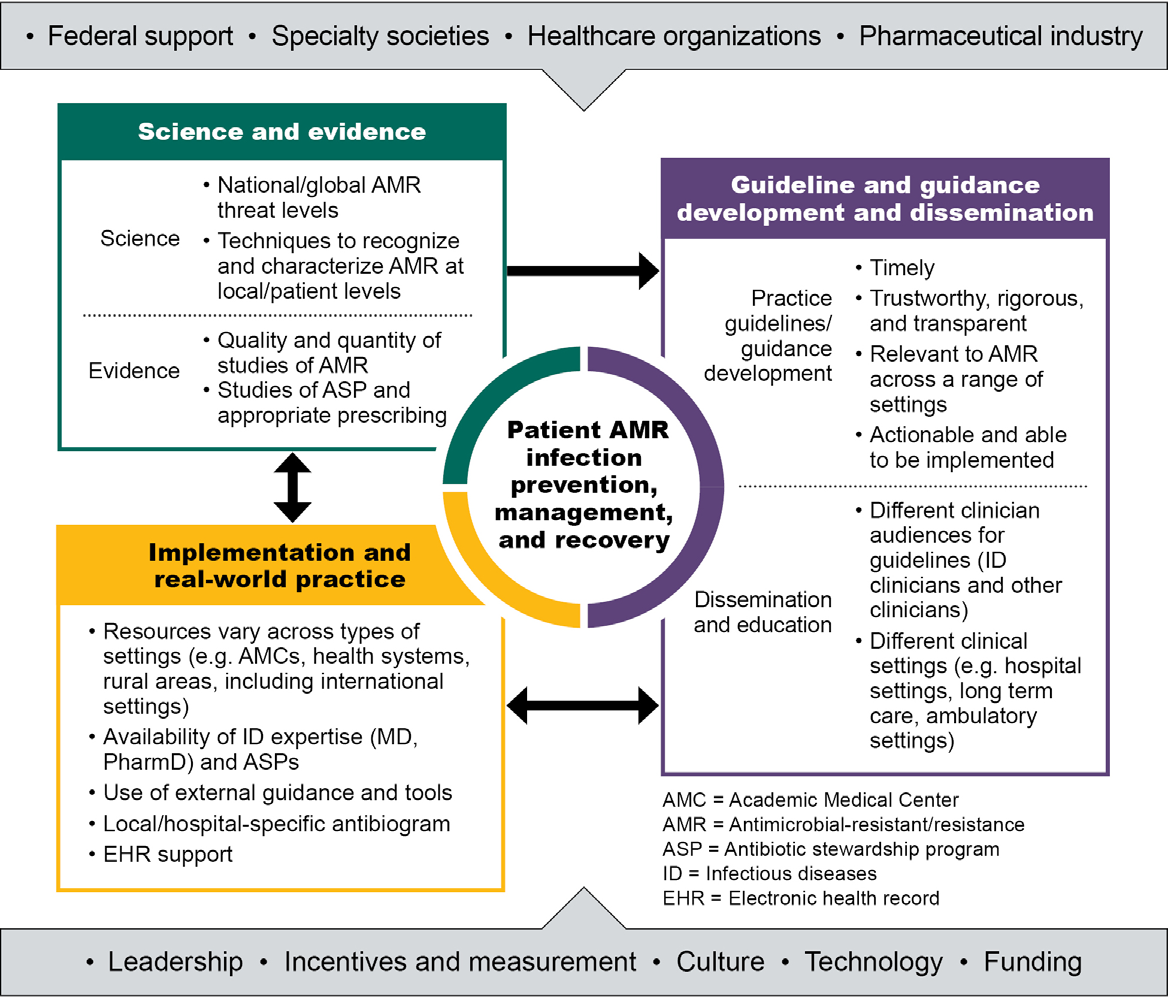
Conceptual framework for guideline development and AMR management.

Stakeholder meeting participants suggested several opportunities to address the challenges related to developing and implementing clinical guidelines. Informed by these suggestions, we developed and revised a conceptual framework for the development and use of clinical practice guidance for the treatment of AMR infections (Fig. 1). We shared a draft of the framework with meeting participants and incorporated their comments.

### Patient and population AMR infection prevention and management

Prevention and management of patients and populations with AMR infections are at the center of the framework, and they are the primary goal of these connected efforts. Recommendations for improving development and use of guidelines should help improve outcomes for the patients and populations for whom the clinical guidelines are relevant.

### Science and evidence inform guideline and guidance development

High-quality scientific evidence drawn from a range of settings and populations is required to inform guideline and guidance development. Scientific knowledge relevant to AMR infections includes epidemiologic descriptions of local, national, and global antimicrobial resistance threats, development of diagnostic techniques to recognize and characterize AMR infections, and discovery and evaluation of novel therapeutics to treat AMR infections. A body of evidence on antimicrobial resistance also includes implementation science studies related to antibiotic stewardship programs and appropriate prescribing patterns.

Evidence for AMR infection prevention and management strategies should be drawn from the different care settings where clinical guidelines are relevant (including not only acute-care hospitals, but also long-term care and ambulatory care settings) to be generalizable to those populations. Additionally, studies should include end points that extend beyond the hospital, such as recurrent infection, readmission, and mortality, to improve understanding of long-term outcomes in nonhospital settings. Science and evidence also influence—and are influenced by—implementation and real-world practice (indicated by the bidirectional arrow from science and evidence to implementation in Fig. 1). Clinical infectious diseases experts and hospital antimicrobial stewardship program leaders often monitor emerging science and evidence and adapt clinical practices in response to new science and evidence before guidelines are updated. Clinical guidelines must also fit into real-world practice, and studies of how to effectively implement guidelines are an important component of the evidence related to clinical guidelines.

### Guideline and guidance development and dissemination

Development and dissemination of practice guidelines and guidance (shown on the right side of the Fig. 1) are shaped by multiple factors. Principles that are important to the development of practice guidelines and guidance include timeliness, trustworthiness and transparency, relevance to management of AMR infections across a range of settings, and ease of implementation. Stakeholders noted that it is important to consider the needs of different clinician audiences and clinical settings in disseminating guidelines and guidance documents.

Strategies to improve timeliness of developing and updating clinical guidelines include using a living guidance approach, as has been done with hepatitis C guidance,^9^ to allow for timely changes as needed. Traditional approaches to guideline development can still be timely when urgency demands sufficient resources. For example, with considerable funding, the IDSA was able to develop comprehensive treatment guidelines for COVID-19 using the GRADE process within 6 weeks.^10^ Although it may be impractical to devote such large resources to every clinical guideline, lessons learned from developing COVID-19 treatment guidelines might be used to develop rigorous guidelines with fewer resources. Specifically, participants noted that GRADE methodologists with expertise and time to support guideline development can help expedite the process. Updates should occur periodically but should be flexible to occur more quickly if groundbreaking studies or the approval of new treatments warrant earlier revision.

Transparency and clarity about the evidence base for the recommendations included in guidelines and guidance documents are essential. Guideline and guidance developers may consider incorporating studies that do not meet GRADE methodological criteria; however, these developers must also clearly communicate the level of evidence informing recommendations, especially in cases where the body of evidence may be less rigorous, or less immediately relevant to clinical practices. Additionally, it may be appropriate to include judgment of experts and leaders in the field where evidence is weak as long as the role of judgment is transparent.

Participants also recommended that, during development, guideline developers should consider how guidelines will be implemented. Assumptions for implementation should be stated in the guidelines, and when the guidelines are released, they could be accompanied by a commentary with additional discussion on implementation. Clinicians’ needs for implementing guidelines or guidance should be understood before starting the development process and should be reassessed after guidelines and guidance are published.

Developers of guidelines and guidance should also consider all relevant care settings. Clinical settings can be heterogenous, for example, a long-term care facility that is part of a large healthcare system may have different resources for implementing guidelines compared to an independent long-term care facility. Guideline and guidance developers may also need to consider how varying ability to implement guidelines or guidance in settings such as indigenous health facilities, rural settings, critical access hospitals, and low- and middle-income countries may shape equitable delivery of care. Guideline developers might present alternative management approaches that are sensitive to differences in resources, staffing levels, and diagnostic capability (eg, access to phenotypic and genotypic tests). For example, guideline developers could incorporate resource-stratified guidelines or guidance for implementing guidelines in settings with more limited access to resources. Guideline developers must also consider different provider audiences who may have different needs. For example, guideline information may be used differently by medical residents in training compared to experienced clinicians or by clinicians with infectious diseases or antibiotic stewardship expertise compared to those in other specialties.

Guideline and guidance documents can be most effective if they can distill the breadth of evidence in an accessible and digestible way, especially for clinicians practicing in care settings outside the hospital such as primary care practices and long-term care facilities. Disseminating guideline summaries, such as through medical association communications, is one way to convey critical information from guidelines to clinicians. EHR integration to allow access to guidelines and enable default order sets that are consistent with guideline recommendations can also improve update of recommendations at the point of care. Other opportunities to make it easier for providers to access the guidelines and guidance at the point of care could be through summary infographics and apps with hospital-specific data such as the hospital’s antibiogram or clinical pathways that translate guidance recommendations into action-oriented processes. Partnering with developers of frequently used clinical resources, such as *UpToDate,* may be another way to broadly disseminate guideline and guidance information.

### Implementation and real-world practice

The ability to implement guidelines or guidance related to AMR infections is influenced by a range of factors including resources (eg, availability of staff with relevant expertise and time), availability of diagnostic tools such as hospital antibiograms, use of other clinical recommendations or clinical protocols, and EHR support. Addressing implementation-related needs in development of guidelines and guidance should be guided by needs assessments and should be a dynamic process that occurs before but also after the guidelines are released. Such a dynamic assessment could help identify whether and how guidelines are used as well as any outstanding questions that can be addressed in guideline updates, or reasons for lack of uptake. Changes in payment policies, such as reimbursed encounters for antibiotic reassessment, may be another way to support guideline-based care.

### Engage stakeholders who shape development and use of guidelines

Key stakeholders who influence guideline and guidance development are noted in the outermost ring of the framework (Fig. 1) and include organizations providing federal support, specialty societies, healthcare organization leaders and clinicians, and the pharmaceutical industry. These organizations and individuals can bring leadership, determine incentives and measurement, shape culture, and provide technology and funding needed to support effective development and use of guidance and guidelines for AMR infections. Although a wide range of stakeholders can influence development and use of guidelines to address AMR infections, improving prevention and management of AMR infections depends on the actions of many. Varying levels of awareness and prioritization, along with the fragmented nature of the healthcare system, present challenges to accelerate change. Engaging leaders and experts, such as those who participated in the interviews and stakeholder meeting that informed this work, is essential to continuing to improve delivery of care related to AMR infections.

Clinical guidelines or guidance documents that provide recommendations for managing AMR infections are an important tool for preventing and mitigating the threat from AMR infections. Findings from key informant interviews and stakeholder discussions provide insights on how to support timely development and use of guidelines and guidance documents for evidence-based management of AMR infections. These findings, summarized in the conceptual framework presented here, include the need for a relevant and robust body of scientific evidence to inform guidelines and guidance; approaches and tools to support timely, transparent guidelines that are relevant and actionable for all clinical audiences; and tools to implement guidelines and guidance effectively. These efforts will require engagement of individuals and organizations with the leadership and leverage needed to support effective development and use of guidance and guidelines for AMR infections.
